# Magnetoliposomes Incorporated in Peptide-Based Hydrogels: Towards Development of Magnetolipogels

**DOI:** 10.3390/nano10091702

**Published:** 2020-08-29

**Authors:** Sérgio R. S. Veloso, Raquel G. D. Andrade, Beatriz C. Ribeiro, André V. F. Fernandes, A. Rita O. Rodrigues, J. A. Martins, Paula M. T. Ferreira, Paulo J. G. Coutinho, Elisabete M. S. Castanheira

**Affiliations:** 1 Centre of Physics (CFUM), University of Minho, Campus de Gualtar, 4710-057 Braga, Portugal; sergioveloso96@gmail.com (S.R.S.V.); raquel.gau@gmail.com (R.G.D.A.); pg37976@alunos.uminho.pt (B.C.R.); pg38822@alunos.uminho.pt (A.V.F.F.); ritarodrigues@fisica.uminho.pt (A.R.O.R.); pcoutinho@fisica.uminho.pt (P.J.G.C.); 2 Centre of Chemistry (CQUM), University of Minho, Campus de Gualtar, 4710-057 Braga, Portugal; jmartins@quimica.uminho.pt (J.A.M.); pmf@quimica.uminho.pt (P.M.T.F.)

**Keywords:** magnetoliposomes, hydrogels, magnetolipogels, self-assembly, fluorescence, Förster resonance energy transfer

## Abstract

A major problem with magnetogels is the encapsulation of hydrophobic drugs. Magnetoliposomes not only provide these domains but also improve drug stability and avert the aggregation of the magnetic nanoparticles. In this work, two magnetoliposome architectures, solid and aqueous, were combined with supramolecular peptide-based hydrogels, which are of biomedical interest owing to their biocompatibility, easy tunability, and wide array of applications. This proof-of-concept was carried out through combination of magnetoliposomes (loaded with the model drug curcumin and the lipid probe Nile Red) with the hydrogels prior to pH triggered gelation, and fluorescence spectroscopy was used to assess the dynamics of the encapsulated molecules. These systems allow for the encapsulation of a wider array of drugs. Further, the local environment of the encapsulated molecules after gelation is unaffected by the used magnetoliposome architecture. This system design is promising for future developments on drug delivery as it provides a means to independently modify the components and adapt and optimize the design according to the required conditions.

## 1. Introduction

Nanomedicine has provided many tools to reduce invasiveness and many acute and chronic side effects associated with chemotherapy while improving patients’ quality of life [1]. The development of new nanosystems has clearly contributed to these advancements. A recent strategy is the combination of liposomes and hydrogels, that might provide better drug formulation stability and drug administration routes [2]. A more robust soft material is attained with the incorporation of magnetic nanoparticles. Such can be obtained, for example, through the combination with magnetoliposomes [3,4]. A different concept is the separate embedding of both nanoparticles and liposomes in the hydrogel matrix [3]. These strategies offer a means of developing multifunctional smart materials that can host membrane-bound enzymes/glycolipids, besides the targeting with a magnetic field gradient and the stimuli-responsiveness through the application of an alternating magnetic field [5]. The on-demand release from stimuli-responsive liposomes enables the use of more potent drugs [6], while the hydrogel immobilizes the components and provides the local environment required to support cell growth [7]. Further, all the components can be independently adjusted, which allows, for example, for the evaluation of which hydrogel is better fitted for a certain composite and application [2]. However, the majority of the developed magnetic liposome-hydrogel complexes have been restricted to the use of polymeric matrices, mainly alginate, and no attention has been given to supramolecular hydrogels.

**Figure 1:**
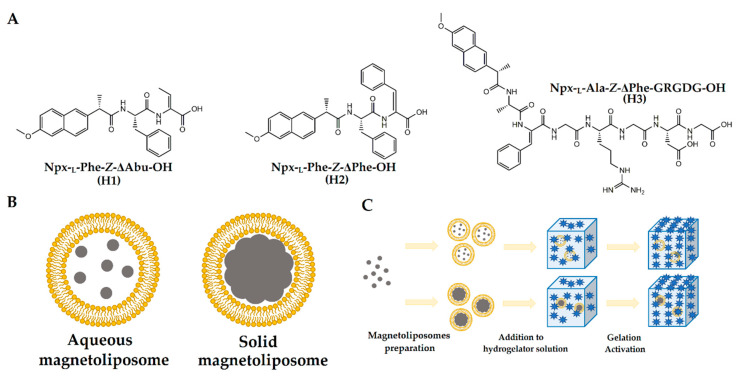
(**A**) Hydrogelator molecules used in this work. Legend: Npx: naproxen; Phe: phenylalanine; Ala: alanine; G: glycine; R: arginine; D: aspartate; ΔAbu: dehydroaminobutyric acid; ΔPhe: Dehydrophenylalanine. (**B**) Schematic representation of the aqueous and solid magnetoliposomes. (**C**) Schematic representation of the strategy used for the development of the supramolecular magnetic liposome-hydrogel complexes.

The self-assembly of supramolecular hydrogelators is driven towards a kinetically trapped intertwined fibrillar structure encompassing solvent pocket microdomains through the cooperative effect of different non-covalent intermolecular interactions [8,9]. The variety of non-covalent intermolecular interactions, both of liposomes and supramolecular hydrogels, might lead to complex behavior and less straightforward magnetic liposome-hydrogel formulation. Hereby, in this work three different hydrogelators (Figure 1A) known to be adequate for drug delivery [10,11] were evaluated as carrying matrixes of magnetoliposomes. Two different types of magnetoliposomes were developed, solid and aqueous, which are schematically represented in Figure 1B. The strategy employed to evaluate this proof-of-concept consisted in preparing the magnetoliposomes, confirming their formation and posterior gelation of the supramolecular hydrogel under the presence of a dilute solution of magnetoliposomes, thus ensuring that the dominant observed effects are exerted by the hydrogel network (or hydrogelator) over the magnetoliposomes membrane (Figure 1C).

## 2. Materials and Methods 

All the solutions were prepared using spectroscopic grade solvents and ultrapure water of Milli-Q grade (MilliporeSigma, St. Louis, MO, USA).

### 2.1. Preparation of Manganese Ferrite Nanoparticles

Manganese ferrite nanoparticles were synthesized by the co-precipitation method, as described in previous works [8,9,12]. Briefly, a mixture of 500 μL of MnSO_4_·H_2_O 0.5 M aqueous solution and 500 μL of FeCl_3_·6H_2_O 1 M was prepared and added, drop by drop, to a 4 mL NaOH 3.4 M aqueous solution at 90 °C, with constant magnetic stirring. After 2 h, nanoparticles purification was carried out by repeated centrifugations, dispersion in deionized water, and drying at 100 °C. The stability of nanoparticles dispersions in PBS medium (pH = 7.0) (with the same nanoparticle concentration used in the preparation of magnetoliposomes) was evaluated by following the UV/Visible absorption for one hour.

### 2.2. Preparation of Magnetoliposomes

For magnetoliposomes preparation, the lipid 1,2-dipalmitoyl-*sn*-glycero-3-phosphatidylcholine (DPPC) (from Sigma-Aldrich, St. Louis, MO, USA), was used. The aqueous magnetoliposomes (AMLs) were developed through the ethanolic injection method [12,13]. Briefly, a 10 mM lipid solution in ethanol was injected, under vigorous agitation, to an aqueous dispersion of magnetic nanoparticles, above the melting transition temperature of DPPC (41 °C) [14]. The mixture was washed with water and purified by magnetic decantation to remove non-encapsulated nanoparticles, as previously reported [15].

Solid magnetoliposomes (SMLs) were developed by a reported method for manganese ferrite nanoparticles [12]. First, 10 µl of a nanoparticle solution (0.02 mg/mL), previously dispersed by sonication at 180 W for one minute, was added to 3 mL of chloroform. After brief sonication and under vigorous agitation, 150 µL of a DPPC 20 mM methanolic solution was added to form the first lipid layer. The first layer-coated nanoparticles were thoroughly washed with water to remove the lipids not attached to the nanoparticles’ surfaces. The nanoparticles were dispersed in 3 mL of water and, under strong agitation, 150 µL of DPPC 20 mM methanolic solution was injected to form the second layer. The resulting solid magnetoliposomes were then washed and purified with ultrapure water by magnetic decantation [12,13]. 

Curcumin and Nile Red were loaded in AMLs through the co-injection method, while in SMLs they were incorporated through the injection of an ethanolic solution upon formation of the second lipid layer [12,13]. 

### 2.3. Spectroscopic and Characterization Measurements

Fluorescence measurements were carried out using a Fluorolog 3 spectrofluorimeter (HORIBA Jobin Yvon IBH Ltd., Glasgow, UK), having double monochromators in excitation and emission, a temperature-controlled cuvette holder and Glan-Thompson polarizers. All fluorescence spectra were corrected for the instrumental response of the system. Absorption spectra were recorded in a Shimadzu UV-3600 Plus UV–Vis–NIR spectrophotometer (Shimadzu Corporation, Kyoto, Japan). 

The mean hydrodynamic diameter, zeta potential and polydispersity index of aqueous and solid magnetoliposomes (lipid concentration: 1 mM) were measured using a NANO ZS Malvern Zetasizer (Malvern Panalytical Ltd., Malvern, UK) dynamic light scattering (DLS) equipment at 25 °C, using a He-Ne laser of λ = 632.8 nm and a detector angle of 173°. Five independent measurements were performed for each sample. High-resolution transmission electron microscopy (HR-TEM) images were obtained in a JEOL JEM 2010F microscope operating at 200 kV (JEOL Ltd., Tokyo, Japan) at C.A.C.T.I (Centro de Apoio Científico e Tecnolóxico á Investigación), Vigo, Spain. A conventional PAN’alytical X’Pert PRO (Malvern Panalytical Ltd., Malvern, UK) diffractometer was used for X-ray diffraction (XRD) analyses, operating with CuK_α_ radiation, in a Bragg–Brentano configuration. Magnetic measurements were performed at room temperature in a Superconducting Quantum Interference Device (SQUID) magnetometer (Quantum Design Inc., San Diego, CA, USA), using applied magnetic fields up to 5.5 T.

### 2.4. Incorporation of the Magnetoliposomes in Hydrogels

All the used hydrogels were prepared for a final concentration of 0.4 wt% (4 mg/mL). Hereby, 1.2 mg of each compound was added to 150 µL of an aqueous solution 2 *v*/*v*% NaOH 1M and dissolved through agitation. After the compound dissolution, the hydrogel solution was taken out of the water bath and mixed with 150 µL of the prepared magnetoliposomes solution. To each mixture, 0.4 wt% of glucono-*δ*-lactone (GdL) was added under agitation, which led to a final pH of ~6–7. The mixture was deployed in a fluorescence microcuvette and left cooling at room temperature, until the hydrogel was formed.

The curcumin release from hydrogels and magnetolipogels (300 µL) loaded with 0.05 mM curcumin was also assessed. The gels containing curcumin were prepared and left stabilizing overnight in Amicon^®^ Ultra-0.5 mL centrifugal filters (MilliporeSigma, St. Louis, MO, USA) with 0.1 µm pore size. Then, pH = 7.0 buffer (800 µL) was added, and the filter tube was immersed and left standing at room temperature. Aliquots were taken after 7 h and fluorescence was measured to determine the concentration. The assays were performed in triplicate.

## 3. Results and Discussion

**Figure 2:**
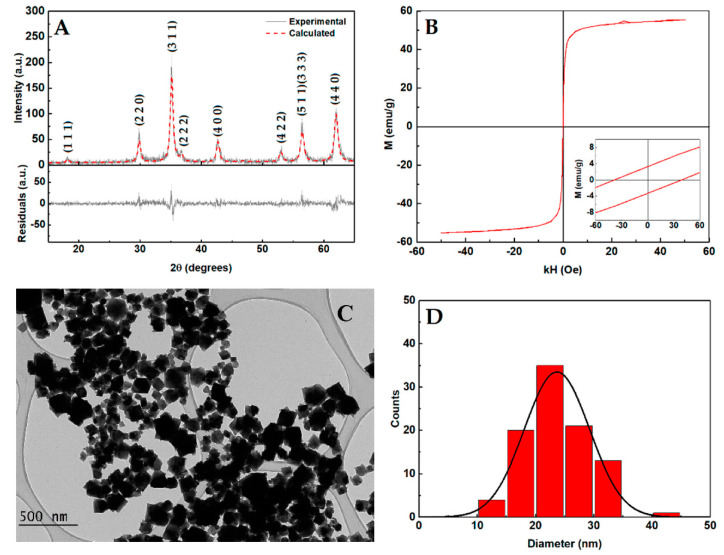
(**A**) X-ray diffraction pattern of manganese ferrite nanoparticles. Grey line: experimental pattern; red line: fitted pattern. (**B**) Magnetization hysteresis loop of the manganese ferrite nanoparticles at room temperature (T = 300 K). Inset: Enlargement of the loop in the low field region. (**C**) Transmission electron microscopy image of the synthesized manganese ferrite nanoparticles. (**D**) Size histogram of the synthesized nanoparticles obtained from TEM.

Manganese ferrite nanoparticles were used for magnetoliposomes development considering their well described synthesis and preparation in the literature [8,9,12,13,16]. The XRD profile is displayed in Figure 2A and confirms the synthesis of a pure crystalline phase of manganese ferrite, as well as presenting all its characteristic peaks, marked by their indices, corresponding to CIF file 2,300,618 (space group Fd-3m:2). The use of a degree of inversion of 0.60, O_x,y,z_ = 0.257 and the micro-absorption correction resulted into a good fitting quality with R_f_ = 3.27 and χ^2^ = 1.19, which is in agreement with the results of manganese ferrite nanoparticles obtained by co-precipitation reported in [16]. An average crystallite size estimate of 12.1 nm was obtained, which is also in close agreement with the sizes reported by Rodrigues et al. of 16.5 nm [12], and 13.3 nm [16], obtained by the same method used in this work. The magnetization hysteresis loop (Figure 2B) displays a saturation magnetization of 55 emu/g, a coercivity of 38.83 Oe, and an M_r_/M_s_ ratio of 0.06, which indicates that the nanoparticles present a superparamagnetic behavior at room temperature, and is in agreement with previously reported values for manganese ferrite nanoparticles obtained through co-precipitation [12,13]. From the TEM measurements (Figure 2C) an average size of 24.2 ± 6.9 nm was obtained, which is also in accordance with previously reported values [9,12].

The stability of nanoparticle dispersions, with and without sonication, was also evaluated. From the UV–Visible absorption measurements over time (Figure S1 in Supplementary Material), it can be observed that nanoparticle dispersions are stable for one hour, with no significant sedimentation and the behavior is similar for nanoparticles with and without sonication. The zeta potential value of the magnetic nanoparticles is negative (−14.1 ± 1.2 mV), preventing their aggregation, as previously reported [9].

The nanoparticles were incorporated into aqueous magnetoliposomes (AMLs), which consist in nanoparticles embedded in the aqueous compartment enclosed by the lipid bilayer, obtained through ethanolic injection of lipids in a well dispersed nanoparticle aqueous solution. The solid magnetoliposomes (SMLs) were obtained as described in previous works, covering a cluster of magnetic nanoparticles with a lipid membrane through a layer-by-layer method [12,13,16]. A Scanning Electron Microscopy (SEM) image of SMLs is exhibited in Figure S2 of the Supplementary Materials. UV–Visible absorbance variations overtime are very low for both AMLs and SMLs (Figure S1 in Supplementary Material), indicating negligible short-time sedimentation. Dynamic light scattering (DLS) results are displayed in Table 1. The aqueous magnetoliposomes of DPPC with entrapped manganese ferrite nanoparticles have diameters of 113.5 ± 10 nm, which is in accordance with those previously reported [12], while DPPC solid magnetoliposomes with sizes around 160 nm were also formerly observed [13]. After one week of storage, the magnetoliposomes remain stable in terms of diameter and surface charge, as inferred from hydrodynamic size and zeta potential values (Table 1), proving the long-term stability of the nanosystems.

The encapsulation efficiency of magnetic nanoparticles in AMLs was determined from the spectrophotometric determination of iron (III) content, through the formation of a phenylfluorone complex sensitized with Triton X-100 (Merck-Sigma, St. Louis, MO, USA), as previously described for other ferrites [15,17]. An encapsulation efficiency of EE(%) ± SD(%) = 74.5 ± 3.5 (from a triplicate assay) compares well with the value previously reported for calcium ferrite (around 70%) [15], being higher than the estimated for iron oxide nanoparticles (EE = 47% ± 15%) [17].

Curcumin was incorporated in AMLs as a model of hydrophobic drugs. Curcumin is a natural polyphenolic compound with various biological activity properties, such as anti-inflammatory, antioxidant and anti-cancer properties, and has been reported to be well encapsulated in magnetoliposomes by Cardoso et al. [14]. Curcumin has also been described to be fluorescent in different polar and non-polar solvents [11,14], and its photophysical behavior is associated with the enol-keto tautomerism of the diketo group, the properties of which are mostly dictated by the enol form (since it is the predominant form in most solvents) [18]. Yet, it has been demonstrated that water stabilizes the diketo form through the formation of stable complexes [19,20]. Fluorescence emission in polar medium is characterized by a large red shift, band enlargement and loss of vibrational structure [14,21].

**Figure 3:**
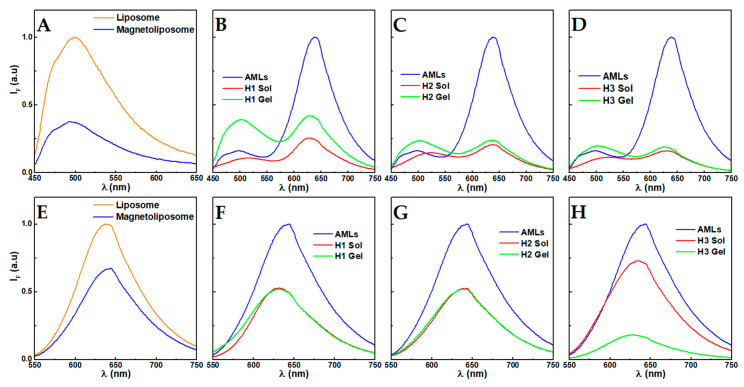
(**A**) Fluorescence emission spectra of curcumin in aqueous magnetoliposomes and liposomes of DPPC (λ_exc_ = 420 nm, [curcumin] = 1 × 10^−6^ M). (**B**–**D**) Fluorescence emission spectra of aqueous magnetoliposomes of DPPC containing curcumin and Nile Red (λ_exc_ = 420 nm, [curcumin] = 1 × 10^−6^ M, [Nile Red] = 1 × 10^−6^ M) in solution of AMLs (AMLs), in a pre-gelation solution (pH ≈ 12, Sol) and hydrogel state (Gel) of the compounds H1, H2 and H3. (**E**) Fluorescence emission spectra of Nile Red in aqueous magnetoliposomes and liposomes of DPPC (λ_exc_ = 520 nm, [Nile Red] = 1 × 10^−6^ M) and (**F**–**H**) in solution of AMLs, in a pre-gelation solution (pH ≈ 12, Sol) and hydrogel state (Gel) of the compounds H1, H2 and H3.

The fluorescence emission spectrum of curcumin in magnetoliposomes is displayed in Figure 3A and compared with the emission in DPPC liposomes. The observed quenching effect in magnetoliposomes may result from an electronic energy transfer to the nanoparticles, as they absorb in a wide energy range [9], as well as due to the heavy-atoms effect, which enhances the efficiency of the intersystem crossing process [22]. The strong fluorescence emission of curcumin is an indication of its presence in the lipid membranes, as it is very weakly emissive in water [11,14]. It has also been reported that curcumin inserts into lipid bilayers in a hydrated environment [23]. Further, the same emission maximum in both liposomes and AMLs (503 nm) indicates that nanoparticles do not affect the membranes.

The hydrogelators used in this work were chosen based on their molecular differences. The hydrogelators H1 and H2 differ in the presence of the aromatic ring in the dehydroamino acid moiety, displaying fibers with an average width of 10 nm (pH ≈ 6) and 14 nm (pH ≈ 8), respectively [10]. The hydrogelator H3 is a linear pentapeptide, with both negative and positive charged groups, and self-assembles into thicker fibers, of 23 nm (pH ≈ 6) width [11].

The lipophilic and solvatochromic probe Nile Red was also included in the aqueous magnetoliposomes to assess any major change in the membrane stability. Its emission blueshifts with the reduction in polarity and is negligible in water, but intensely emits in non-polar environments [24,25,26,27]. Furthermore, the fluorescence risetime is sensitive to viscosity, owing to an activation barrier required for the formation of a twisted intramolecular charge transfer state (TICT) [28,29].

The Förster resonance energy transfer (FRET) process between curcumin (energy donor) and Nile Red (energy acceptor) that has been used in different works as the spectral overlap between the Nile Red absorption and curcumin fluorescence is significant [9,11,30]. Figure 3B–D display the FRET process between curcumin and Nile Red, as evidenced by the strong fluorescence emission of Nile Red, while exciting the curcumin dye (λ_exc_ = 420 nm). Before mixing the AMLs and hydrogelator solutions, curcumin displays its maximum around 500 nm, indicating an environment similar to chloroform [11], and characteristic of its incorporation in DPPC membranes (i.e., Nile Red co-encapsulation did not affect curcumin microenvironment) [31], while the Nile Red maximum is at ~638 nm. After the addition of the AMLs to the hydrogelator solutions, and before inducing gelation (2 *v*/*v*% NaOH 1 M), a decrease in the fluorescence emission and FRET efficiency is clearly observed. Besides the scattering associated with the presence of the hydrogelator micelles, such can also be a consequence of the deprotonation of curcumin. The formation of spherical or worm-like micelles by *N*-capped dipeptides at high pH has been reported by Cardoso et al. [32]. The curcumin deprotonation is further evidenced by the red-shift to λ ~ 530 nm. The high pH induces the deprotonation of the three hydroxyl groups of curcumin (pK_a_ values are 8.38, 9.88 and 10.51) and, consequently, its solubility is slightly improved [33,34]. Furthermore, while the neutral form is preferentially located in the hydrophobic phase, at basic pH it accumulates in the surface of the lipid bilayer [35]. Thus, while some curcumin is expected to remain in the inner cavity or membrane of the AMLs, some might relocate to the outer surface when the pH is increased, which leads to its release towards the aqueous phase and a larger distance from Nile Red. This potential outcome is also evidenced by the reduction in FRET efficiency upon pH decrease when the gel state is attained. In this state, a blue-shift of curcumin fluorescence emission is associated with its protonation and relocation or adsorption to hydrophobic cavities, both in the hydrogel matrix and hydrophobic phase of the liposomes, as suggested by both the reduced FRET efficiency and a slight increase in Nile Red emission.

The direct excitation of Nile Red provides further information on the membrane dynamics. Overall, a decrease in fluorescence intensity is observed in the concentrated hydrogelator solutions containing the AMLs. The decrease is similar in the gels of compounds H1 and H2, which remains unchanged after gelation, while the H3 gel displays a strong fluorescence decrease after gelation. Furthermore, in the gels H1 and H3, a slight blue-shift occurs from 640 nm to 631 nm and 628 nm, respectively. This suggests an interaction between the dehydrodipeptides and the AMLs membrane, leading to a higher hydrophobicity of the membrane region where Nile Red is localized. The stronger decrease in the H3 gel after gelation can be associated with the larger diameter of the fibers increasing the inner filter effect, considering that the solution changed from a translucid solution to a turbid gel.

**Figure 4:**
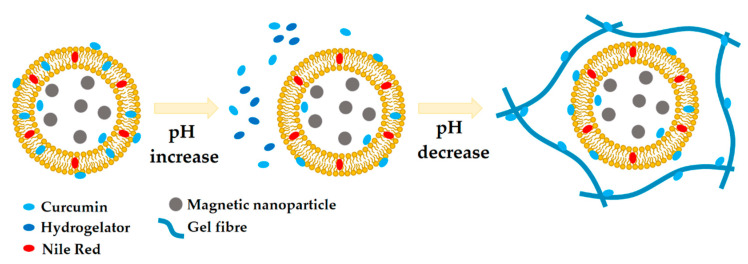
Scheme of the proposed process of incorporation of AMLs containing curcumin (that becomes more water soluble at high pH) and Nile Red in a supramolecular dehydrodipeptide hydrogel activated through GdL-induced slow pH decrease. As the pH is increased, some of the curcumin molecules are dissolved and, once the pH is decreased, it adsorbs and relocates to the hydrophobic cavities of both the hydrogel fibers and AMLs membranes.

The scheme in Figure 4 summarizes the observed behavior and the expected mechanism of encapsulation of hydrophobic drugs (e.g., curcumin, and Nile Red as a lipid probe), upon the preparation of magnetic lipogels bearing AMLs, though it is pointed out that curcumin becomes more hydrophilic at high pH [20]. Further, considering the AMLs membranes as mimetics of the biological membranes, it is expected that the in situ gelation of supramolecular gels will not lead to membrane disruption. Nonetheless, it cannot be ignored that the observed partition may result from a potential membrane perturbation by the encapsulated molecules. Curcumin is known to modify the lipid bilayer properties, such as bilayer stiffness, thickness, elasticity moduli, and curvature [36,37,38]. For instance, it was reported to decrease the membrane stiffness in the absence of cholesterol [39]. The increase in membrane flaccidity might also favor the redistribution of both curcumin and Nile Red between the membranes and the hydrogel fibers.

**Figure 5:**
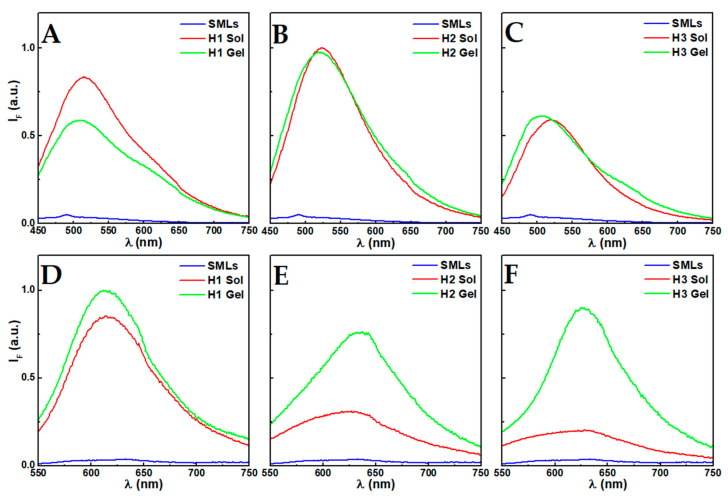
(**A**–**C**) Fluorescence emission spectra of curcumin and Nile Red in solid magnetoliposomes of DPPC (λ_exc_ = 420 nm, [curcumin] = 2 × 10^−6^ M, [Nile Red] = 2 × 10^−6^ M) in solution of SMLs (SMLs), in a pre-gelation solution (pH ≈ 12, Sol) and hydrogel state (Gel) of the compounds H1, H2 and H3. (**D**–**F**) Fluorescence emission spectra of Nile Red (λ_exc_ = 520 nm, [Nile Red] = 2 × 10^−6^ M) in solid magnetoliposomes in an aqueous solution (SMLs), in a pre-gelation solution (pH ≈ 12, Sol) and hydrogel state (Gel) of the compounds H1, H2 and H3.

Solid magnetoliposomes (SMLs) were prepared with the co-encapsulation of both curcumin and Nile Red, where the former was demonstrated in previous works to be finely encapsulated in these systems [14,21]. The fluorescence emission spectra of the aqueous solution of SMLs, after addition to the concentrated basic hydrogelator solutions and gelation, are displayed in Figure 5. The strong fluorescence quenching is a consequence of the proximity of both molecules to the cluster of magnetic nanoparticles. Both curcumin (maximum around 500 nm—i.e., similar microenvironment to liposomes and AMLs) and Nile Red (at 631 nm) fluorescence emission maxima are associated with their location in the membranes. Upon addition of the SMLs to the concentrated hydrogelator solution, a strong enhancement of curcumin emission is observed, which might be a result of the localization of curcumin from the SMLs membranes towards the hydrogelator micelles that display a more hydrated environment, as suggested by the red-shift (H1: 521 nm; H2: 524 nm; H3: 526 nm). Upon pH decrease, similar to what was described in the AMLs incorporation, curcumin accumulated either in the hydrogel fibers or in the SMLs membranes.

In H1 (510 nm) and H3 (511 nm) systems, curcumin is localized in an environment with a polarity similar to chloroform, while in H2 (521 nm) the emission is only slightly blue-shifted, indicative of an environment with a polarity similar to acetonitrile [11]. The fluorescence quenching of H1 suggests its proximity to the SMLs. The localization of curcumin in a hydrophobic environment has been reported in a previous work with magnetic gels [9]. Yet, the presence of membranes favored the location of curcumin to a more hydrophobic environment in the H3 gel, as opposed to the previously reported environment similar to acetonitrile in the magnetic gels. Further, after gelation, an increase around 630 nm is observed, which is associated with the occurrence of FRET between curcumin and Nile Red.

Similar to curcumin behavior, Nile Red fluorescence emission is unquenched and some changes in the environment occur upon mixture with the hydrogelator solutions (Figure 5). In the H1 gel, a redshift to 620 nm was obtained, while in H2 and H3 the maximum wavelength remained centered around 631 nm. The enhancement of Nile Red and curcumin fluorescence after gelation suggests that more hydrophobic cavities are made available to accommodate the molecules from the previously highly saturated magnetoliposomes—i.e., the curcumin and Nile Red release from the SMLs towards the hydrogels was favored.

**Figure 6:**
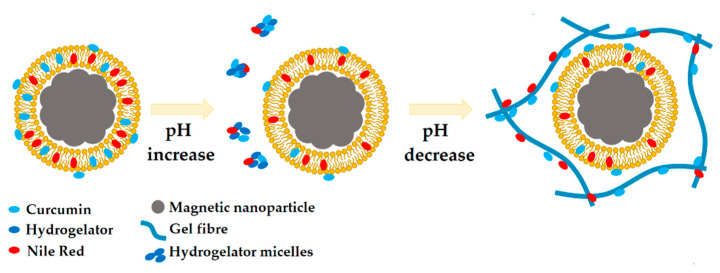
Scheme of the proposed process of incorporation of SMLs containing curcumin (that becomes more water soluble at high pH) and Nile Red in a supramolecular dehydrodipeptide hydrogel activated through GdL-induced slow pH decrease. Upon combination of the SMLs with the basic pH hydrogelator solution, both curcumin and Nile Red are released and accumulate in the hydrophobic cavities of the hydrogelator micelles. As the pH is lowered, both molecules can accumulate in both the hydrophobic cavities of the hydrogel matrix and the SMLs membrane.

The scheme included in Figure 6 summarizes the discussed process and the expected behavior of hydrophobic drugs (curcumin is sensitive to pH variations) encapsulated in SMLs upon the preparation of supramolecular magnetic lipogels through slow pH decrease by GdL. The observed behavior provides a means to ensure that no premature release of the administered drugs from the SMLs occurs, as they are retained by the hydrogel matrix or micelles. Moreover, it makes the co-delivery or incorporation of larger amounts of hydrophobic drugs possible, as the hydrogel matrix develops hydrophobic cavities capable of accommodating the excessive amount from the saturated SMLs. For instance, a lower percentage of curcumin release after 7 h of incubation with pH = 7.0 buffer was obtained in both magnetolipogels (Figure S3 in Supplementary Material). These results point out that the presence of magnetoliposomes in the hydrogel matrix can reduce the release of curcumin.

Steady-state fluorescence anisotropy measurements were carried out to evaluate the effect of the hydrogel on the microviscosity of Nile Red local environment. Table 2 displays the obtained values for both AMLs and SMLs before and after gelation through the pH trigger.

In all systems, the fluorescence anisotropy (and, thus, the local microviscosity) increases when compared to the neat magnetoliposomes (Table 2). Further, the anisotropy converges to close values in both systems, which suggests that similar interactions are established between the hydrogel matrix and both magnetoliposome architectures. This convergence on microviscosity offers the possibility of exploring different systems to optimize therapeutic strategies, as the distribution and behavior of amphipathic and hydrophobic drugs are expected to be similar to the distribution of the molecules here discussed. For instance, the presence of AMLs allows for the delivery of hydrophilic drugs in the aqueous cavity, besides the delivery of hydrophobic drugs in the membrane and amphipathic drugs distributed along both the hydrogel matrix and the magnetoliposome, while the incorporation of SMLs allows for the delivery of hydrophobic and amphipathic drugs at higher concentrations than the single use of SMLs, without severely affecting the magnetization (and, consequently, hyperthermia) of the magnetic nanoparticles, as the amount of diamagnetic mass is reduced.

## 4. Conclusions

In this work, the incorporation of solid and aqueous magnetoliposomes in supramolecular hydrogels was assessed in three different gels through a pH trigger. It was demonstrated that, upon the incorporation of the magnetoliposomes, the encapsulated molecules distributed to a similar environment independently of the magnetoliposome architecture. Further, magnetoliposomes can be saturated with the required drug, as the hydrogel displays hydrophobic cavities that accommodate the excessive drug amount, which can be explored as a way to avert the premature release of the administered drug. This system design approach provides a useful strategy to increase the array of drugs that can be encapsulated compared to magnetic gels and magnetoliposomes alone. Concerning the discussed findings in this communication, research is being carried out to assess the influence of the different magnetic lipogel architectures on drug delivery control and tunability, as well as to evaluate its impact on hyperthermia capability.

## Figures and Tables

**Table 1:** Hydrodynamic size, zeta potential and polydispersity index values of aqueous and solid magnetoliposomes based on manganese ferrite nanoparticles, immediately after preparation and after one week of storage (SD: standard deviation; PDI: Polydispersity index).

	Size ± SD (nm)		PDI ± SD		Zeta Potential ± SD (mV)	
	After Preparation	1 Week After	After Preparation	1 Week After	After Preparation	1 Week After
AMLs	113.5 ± 10	98.7 ± 17	0.23± 0.04	0.22 ± 0.08	−15.3 ± 2	−16.1 ± 4
SMLs	156.3 ± 16	132.2 ± 21	0.25 ± 0.03	0.21 ± 0.07	−21.4 ± 4	−19.7 ± 3

**Table 2:** Steady-state fluorescence anisotropy (*r*) values of Nile Red for gels with the incorporated AMLs or SMLs. Values in neat magnetoliposomes (MLs) are shown for comparison.

System	MLs	H1 Sol	H1 Gel	H2 Sol	H2 Gel	H3 Sol	H3 Gel
AMLs	0.20	0.17	0.26	0.27	0.28	0.22	0.26
SMLs	0.06	0.22	0.24	0.16	0.25	0.15	0.26

## Supplementary Materials

The following are available online at https://www.mdpi.com/2079-4991/10/9/1702/s1, Figure S1: Stability of Nanoparticles and Magnetoliposomes Dispersions, Figure S2: SEM Image of Solid Magnetoliposomes, Figure S3: Curcumin Release Assay.
