# Supplementary material for: Magnetoliposomes Incorporated in Peptide-Based Hydrogels: Towards Development of Magnetolipogels
Source: Nanomaterials (Basel). 2020 Aug 29;10(9):1702. doi: 10.3390/nano10091702 (PMC7558371; doi:10.3390/nano10091702)
Supplement: Supplementary file 1 [file nanomaterials-10-01702-s001.pdf]

Supplementary Materials

Magnetoliposomes Incorporated in Peptide-Based Hydrogels: Towards Development of Magnetolipogels

Sérgio R. S. Veloso [1], Raquel G. D. Andrade [1], Beatriz C. Ribeiro [1], André V. F. Fernandes [1], A. Rita O. Rodrigues [1], J. A. Martins [2], Paula M. T. Ferreira [2], Paulo J. G. Coutinho [1] and Elisabete M. S. Castanheira [1,*]

[1] Centre of Physics (CFUM), University of Minho, Campus de Gualtar, 4710-057 Braga, Portugal; sergioveloso96@gmail.com (S.R.S.V.); raquel.gau@gmail.com (R.G.D.A.); pg37976@alunos.uminho.pt (B.C.R.); pg38822@alunos.uminho.pt (A.V.F.F.); ritarodrigues@fisica.uminho.pt (A.R.O.R.); pcoutinho@fisica.uminho.pt (P.J.G.C.)

[2] Centre of Chemistry (CQUM), University of Minho, Campus de Gualtar, 4710-057 Braga, Portugal; jmartins@quimica.uminho.pt (J.A.M.); pmf@quimica.uminho.pt (P.M.T.F.)

* Correspondence: ecoutinho@fisica.uminho.pt; Tel.: +351-253-604-320

Stability of nanoparticles and magnetoliposomes dispersions

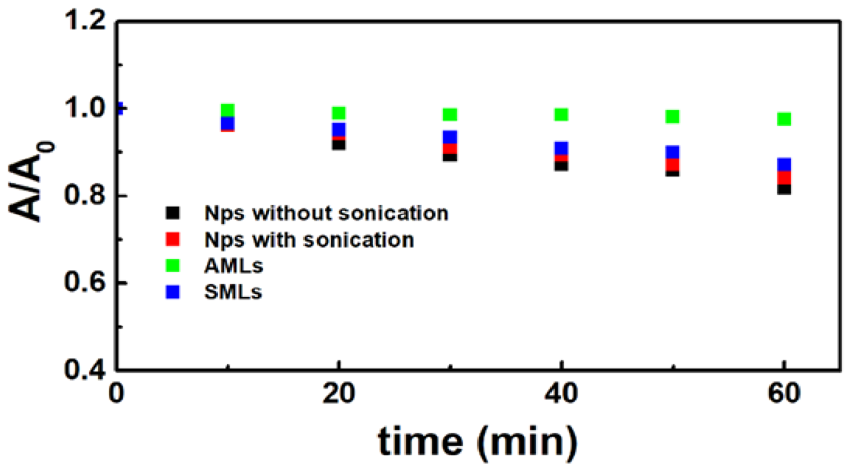

Figure S1. Variation of UV-Visible absorbance of nanoparticles (Nps) dispersions (with and without sonication), aqueous magnetoliposomes (AMLs) and solid magnetoliposomes (SMLs) in PBS buffer (pH = 7.0) as function of time, for 1 h.

SEM image of solid magnetoliposomes

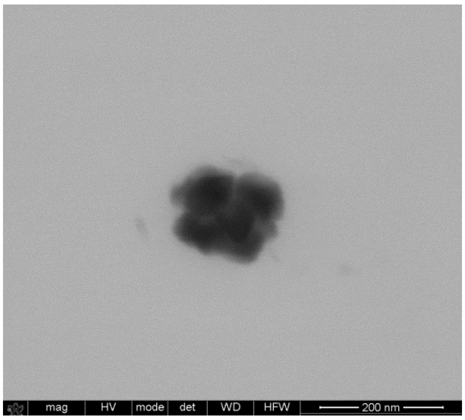

Figure S2. Scanning Electron Microscopy (SEM) image of solid magnetoliposomes (SMLs).

Curcumin release assay

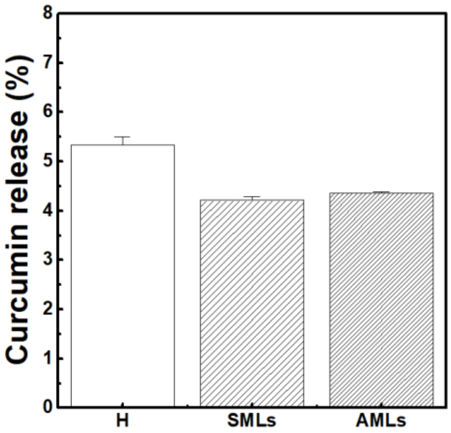

Figure S3. Percentage of curcumin release from the hydrogel (H) and gels containing aqueous (AMLs) and solid (SMLs) magnetoliposomes in PBS buffer (pH = 7.0) after 7 h.
